# Spinal cannabinoid receptor 2 activation reduces hypersensitivity associated with bone cancer pain and improves the integrity of the blood–spinal cord barrier

**DOI:** 10.1136/rapm-2019-101262

**Published:** 2020-08-12

**Authors:** Chenchen Wang, Ke Xu, Yu Wang, Yanting Mao, Yulin Huang, Ying Liang, Yue Liu, Jing Hao, Xiaoping Gu, Zhengliang Ma, Yu'e Sun

**Affiliations:** Department of Anesthesiology, Nanjing University Medical School Affiliated Nanjing Drum Tower Hospital, Nanjing, Jiangsu, China

**Keywords:** animal studies, basic science of pain, pharmacology: other

## Abstract

**Background:**

Disruption of the blood–spinal cord barrier (BSCB) can facilitate inflammation that results in pain hypersensitivity. Proinflammatory cytokines produced by activated microglia and astrocytes damage the BSCB. This study aims to explore whether the BSCB is damaged in the bone cancer pain (BCP) model and to investigate a potential role and mechanism of JWH015 ((2-methyl-1-propyl-1H-indol-3-yl)−1-naphthalenylmethanone), a selective cannabinoid receptor 2 (CB2R) agonist, in preserving the BSCB integrity in the BCP model.

**Methods:**

We used a male mouse model of BCP. Pain hypersensitivity was measured over time. Evans blue dye extravasation, transmission electron microscopy and Western blotting were performed to investigate the permeability and structural integrity of the BSCB. Immunofluorescence staining and western blotting were used to investigate the effect of JWH015 on the activation of glial cells and the levels of proinflammatory cytokines.

**Results:**

A single intrathecal injection of JWH015 ameliorated pain hypersensitivity, the BSCB disruption and microglia and astrocyte activation. Decreases in the expression of ZO-1 and claudin-5 were partially restored by JWH015. The levels of the proinflammatory cytokines interleukin-1β and tumor necrosis factor-α and the enzyme MMP9 were reduced by JWH015. However, all effects were prevented by pretreatment with a CB2R-selective antagonist, AM630 ((6-iodo-2-methyl-1-(2-morpholinoethyl)−1H-indol-3-yl)(4-methoxyphenyl)methanone).

**Conclusions:**

JWH015 alleviates neuroinflammation and maintains the BSCB integrity and permeability in a mouse model of BCP, which is probably mediated by inhibiting glial cells activation. This study reveals the new analgesic mechanism of JWH015 on BCP and provides a perspective to explore novel drugs that target the BSCB to control BCP.

## Introduction

Bone cancer pain (BCP) is a severe complication of metastatic or advanced malignancy, which is characterized by allodynia and hyperalgesia.^1^ BCP occurs in approximately 21% of patients who die of cancer, which decreases the patient’s quality of life and imposes a substantial burden on society.^2^ The mechanism underlying BCP is not yet clear, and consequently, the efficacy and side effects of the existing drug interventions are still not satisfactory.^1^

The endocannabinoid system consists of cannabinoid receptors (CBR), their ligands and the enzymes required for the synthesis and degradation of cannabinoids.^3^ CBR 2 (CB2R) is expressed primarily in the immune system.^4^ The analgesic effects of CB2R agonists on a number of pain models have been observed,^5 6^ but a definitive explanation of the various analgesic mechanisms has not yet been attained. CB2R activation was recently shown to be associated with the suppression of blood–brain barrier (BBB) damage. CB2R-selective agonist O-1966 ((1-[4-(1,1-dimethyl-heptyl)−2,6-dimethoxy-phenyl]−3-methyl-cyclohexanol)) and JWH133 ([(6aR,10aR)−3-(1,1-dimethylbutyl)−6a,7,10,10a-tetrahydro-6,6,9-trimethyl-6H-dibenzo[b, d]pyran]) prevent BBB leakage in subjects with traumatic brain injury^7^ and Parkinson’s disease,^8^ respectively.

The BBB including the blood-spinal cord barrier (BSCB), is primarily formed by capillary endothelial cells that are surrounded by the basal lamina, pericytes and astrocytic endfeet. The presence of tight junctions (TJ) between endothelial cells forms a tight seal to limit the entry of neurotoxic plasma components, blood cells and pathogens into the brain or spinal cord.^9^ ZO-1 and claudin-5 are two representative TJ proteins whose loss has been verified to induce BBB breakdown in many neurodegenerative disorders and acute CNS diseases, such as Alzheimer’s disease and spinal cord injury.^9^ According to recent studies, prolonged BSCB dysfunction facilitates the infiltration of peripheral leukocytes into the spinal parenchyma to amplify nociceptive signaling in chemotherapy-induced neuropathic pain and peripheral neural injury-induced inflammatory pain.^10 11^ Therefore, the present study aimed to determine whether the analgesic effect of the CB2R-selective agonist JWH015 on BCP was mediated by improving the damaged BSCB.

Increased inflammation and oxidative stress have been shown to increase the BBB permeability, accompanied by a loss of TJ proteins.^12^ Proinflammatory cytokines with excessive activity, such as tumor necrosis factor-α (TNF-α), interleukin-1β (IL-1β) and the enzyme MMP9 disrupt the BBB by altering the levels or function of TJ proteins.^13 14^ Activated microglia and astrocytes produce IL-1β and TNF-α to elicit central sensitization and facilitate BCP,^15^ and release MMP9 to maintain neuropathic pain.^16^ However, no study has explored the role of MMP9 in BCP. In our previous study, JWH015 produced antinociceptive effects by suppressing the activation of glial cells and proinflammatory cytokine production in postoperative pain.^5^ CB2R activation induces an anti-inflammatory phenotype of microglia to resolve neuropathic pain.^6^ It was hypothesized that JWH015 improved the permeability of the BSCB by inhibiting neuroinflammation in BCP.

In this study, we hypothesized that spinal CB2R activation ameliorated BSCB damage induced by glia-derived proinflammatory factors and attenuated pain hypersensitivity associated with BCP. Therefore, we tested the hypothesis by detecting the activation of spinal glia, the levels of proinflammatory factors and the integrity of the BSCB in a mouse model of BCP, and subsequently examined the effect of JWH015.

## Materials and methods

### Animals

Male C3H/HeN mice (weighing 20–25 g; aged 5 weeks; Vital River Experimental Animal Corporation of Beijing; n=223) were housed in the same room at a constant temperature (22°C±2°C) and humidity (55%±5%) and maintained on a 12:12 hours light-dark cycle with free access to water and food. The mice were randomly allocated to the following groups: (1) control, (2) tumor group, (3) sham group, (4) tumor+vehicle group, (5) tumor+JWH015 group, (6) tumor+AM630+JWH015 group. Eight mice were assigned to each group for behavior test and three mice were assigned to each group at various time points for the molecular testing.

### Animal model of BCP

NCTC 2472 sarcoma cells were cultured in NCTC 135 medium (Sigma, USA) containing 10% horse serum (Gibco, USA) at 37°C in a 5% CO_2_ incubator (Thermo Forma, USA).

The BCP model was established using the method described by Schwei *et al*.^17^ Mice were anesthetized with an intraperitoneal injection of pentobarbital sodium at a dose of 50 mg/kg, and an arthrotomy was performed on the right knee. Next, 20 µL of α–Minimum Essential Medium (α-MEM) (Thermo Fisher Scientific, USA) containing 2×10^5^ NCTC 2472 cells or α-MEM without cells were injected into the intramedullary cavity of the right distal femur, and designated the tumor group of mice and sham group of mice, respectively. After sealing the injection hole and closing the wound, mice were resuscitated on a heated blanket.

### Drugs and treatments

The CB2R agonist JWH015 (2 µg; sigma, USA) and antagonist AM630 (1 µg; sigma, USA) were dissolved in 5 µL of 20% dimethyl sulfoxide (DMSO) (in normal saline). On day 14 after sarcoma cells implantation, mice in the tumor+JWH015 group received a single intrathecal injection of 2 µg of JWH015. Mice in the tumor+AM630+JWH015 group received a single intrathecal injection of 1 µg of AM630 30 min before the injection of JWH015. Mice in the tumor+vehicle group received the same volume of 20% DMSO under identical conditions as the control. Doses of these compounds were based on our previous study.^18^

### Nociceptive behavioral tests

Mechanical allodynia and spontaneous pain were tested before the operation (day 0) and on days 4, 7, 10, 14 and 21 after the operation in the tumor group of mice and sham group of mice, before the injection (0 hour) and at 2, 6, 12, 24, 48 and 72 hours after the injection in the tumor+vehicle group, tumor+JWH015 group and tumor+AM630+JWH015 group. All nociceptive behavioral tests were performed by an experimenter who was unaware of the group assignments.

The paw withdrawal mechanical threshold (PWMT) of the hind paw was measured using von Frey filaments (0.16, 0.4, 0.6, 1.0, 1.4 and 2.0 g; Stoelting, USA), as previously reported.^18^ After a 30 min acclimation period, von Frey filaments were poked vertically into the plantar surface and the lowest stimulus strength that caused paw brisk withdrawal or flinching reflexes was regarded as PWMT.

The number of spontaneous flinches (NSF) of the hind paw was quantified over 2 min after a 30 min acclimation period. Every lift of the hind paw that was not associated with walking or grooming was regarded as one flinch.

### Assessment of the permeability of the BSCB

Evans Blue dye extravasation was analyzed to investigate the integrity of the BSCB using a previously reported method,^19^ with a few modifications. Evans blue dye (2% w/v in saline, 4 mL/kg, Sigma-Aldrich, USA) was injected into the tail vein of the mouse. Two hours later, mice were anaesthetized and perfused with saline through the left ventricle. The lumbar enlargement of spinal cord was harvested, weighed and homogenized in formamide. After centrifuging, supernatants were collected and fluorescence was quantified at an excitation wavelength of 620 nm. The amount of dye in the samples was calculated as μg/g of tissue using a standard curve.

### Transmission electron microscopy

After anesthetization, mice were perfused with normal saline. The lumbar enlargements were harvested and incubated with 2.5% glutaraldehyde. Samples were post-fixed with 1% osmium tetroxide and dehydrated in a graded series of diluted acetone solutions. After embedding in epoxy resin, samples were cut into ultrathin sections and stained with uranyl acetate and citric acid lead. Images were captured using a Hitachi 7700 transmission electron microscope.

### Western blotting analysis

Mice were sacrificed under deep anesthesia. Total protein from the entire spinal cord lumbar enlargements were extracted using ice-cold RIPA Lysis Buffer. Protein samples were separated on SDS-PAGE gels and then transferred onto a polyvinylidene fluoride membrane (Millipore, USA). After blocking with skim milk, the membranes were incubated with the following primary antibodies overnight at 4℃: ZO-1 (1:1000, Thermo Fisher, USA), Claudin-5 (1:1000, Thermo Fisher, USA), Iba1 (1:700, Wako, Japan), GFAP (1:2000, CST, USA), TNF-α (1:200, Santa Cruz, USA), IL-1β (1:1000, Abcam, USA), MMP9 (1:1000, Abcam, USA), CB2R (1:1000, Bioworld, China) and Tubulin (1:1000, Bioss, China). After washes with TBST and an incubation with the secondary antibody (1:10000, Millipore, USA), membranes were visualized using ECL solution. Images were captured and analyzed using a cooled CCD system (Tanon, China).

### Immunofluorescence staining

After general anesthesia was administered, mice were intracardially perfused with normal saline followed by 4% paraformaldehyde. The lumbar enlargements were removed, post-fixed with 4% paraformaldehyde and then dehydrated in 30% sucrose. Samples were cut into 20 µm sections using a freezing microtome. After washes with phosphate buffer saline (PBS), the sections were blocked with 10% goat serum containing 0.3% Triton X-100 and then incubated with the following primary antibodies in 10% goat serum overnight at 4℃: Iba1 (rabbit, 1:500, Wako, Japan), GFAP (mouse, 1:500, CST, USA), CB2R (rabbit, 1:100, Abcam, USA) and OX42 (mouse, 1:500, Abcam, USA). After washes with PBS, sections were incubated with secondary antibodies in 10% goat serum, including an Alexa 488-conjugated goat anti-rabbit antibody (1:3000, Thermo Fisher, USA) and Alexa 594-conjugated goat anti-mouse antibody (1:3000, Thermo Fisher, USA). Then, sections were washed with PBS, transferred to glass slides, air-dried and stained with 4’,6-Diamidino-2-Phenylindole (DAPI) (Abcam, USA). Images were captured using a laser-scanning confocal microscope (Olympus, Japan). Three sections from three mice from each group were analyzed.

### Statistical analyses

The number of mice used in each study was based on our previous experience with this design.^20^ No a priori statistical power calculation was used to guide the sample size selection. Data are presented as the means±SD. SPSS V.20 (IBM) was used for the statistical analysis. Differences in nociceptive behaviors at each time point between groups were analyzed using multiple comparisons and changes in nociceptive behaviors over time within groups were assessed using repeated measurements analysis of variance (ANOVA). One-way ANOVA was performed to determine differences in the results of western blotting and immunofluorescence staining among groups. Bonferroni’s post hoc tests were used to detect the significance of the differences. All tests were two tailed, and p<0.05 was considered statistically significant.

## Results

### JWH015 alleviates mechanical hyperalgesia in BCP mice over time

**Figure 1:**
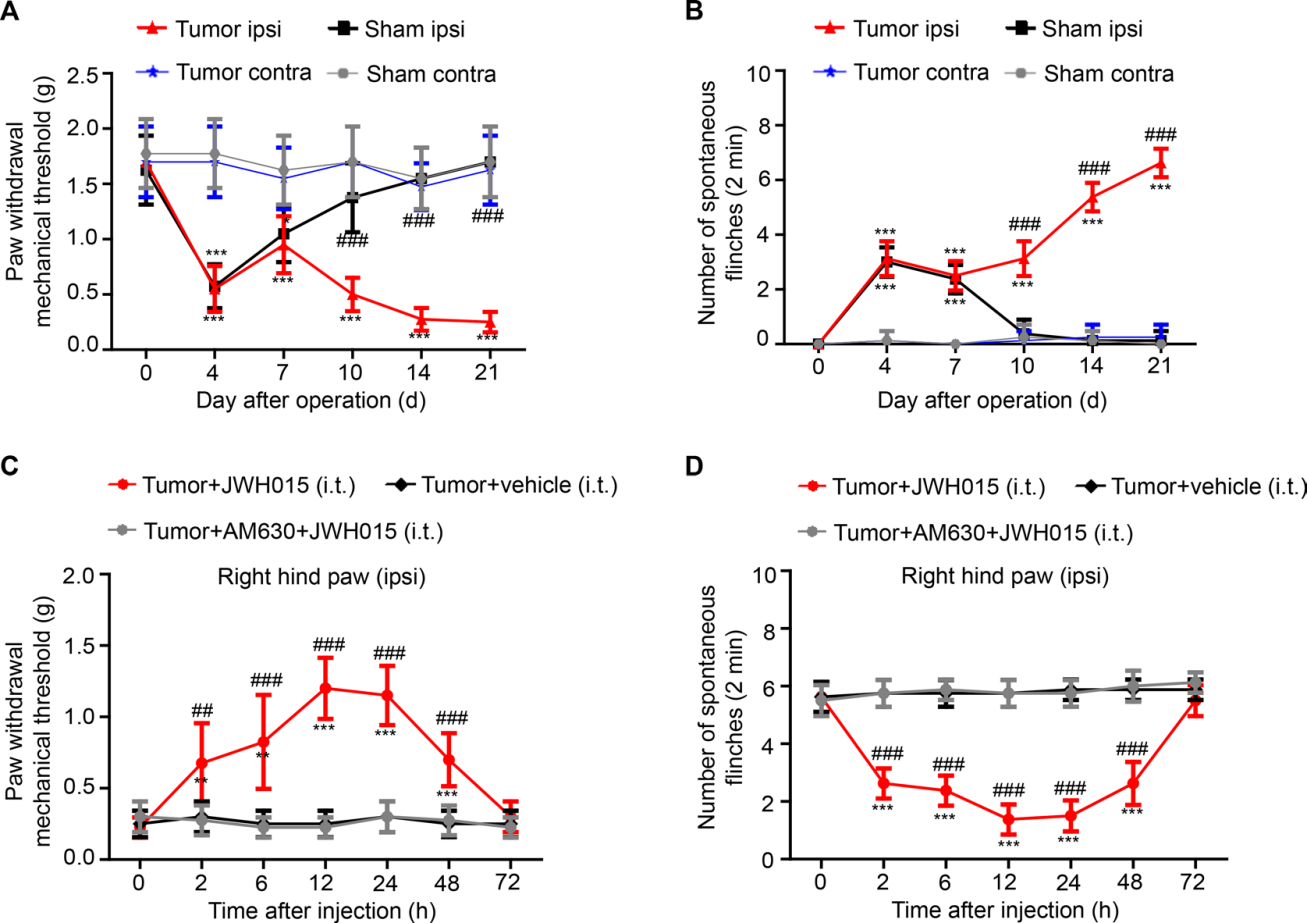
Pain hypersensitivity over time. (A) The PWMT and (B) the NSF were tested on days 0, 4, 7, 10, 14 and 21 after operation in the sham and tumor groups. *P<0.05 vs day 0, **p<0.01, ***p<0.001; ^#^p<0.05 vs the sham group at each point, ^##^p<0.01, ^###^p<0.001; n=8 per group. Pain hypersensitivity was alleviated in a time-dependent manner after a single intrathecal injection of JWH015. (C) The PWMT and (D) the NSF were tested before the injection (0 hour) and at 2, 6, 12, 24, 48 and 72 hours after the injection of JWH015, vehicle and AM630+JWH015 in the tumor group. *P<0.05 vs preinjection, **p<0.01, ***p<0.001; ^#^p<0.05 vs the tumor+vehicle group at each point, ^##^p<0.01, ^###^p<0.001; n=8 per group. All data are presented as the means±SD. Contra, contralateral hind paw to the surgery; ipsi, ipsilateral hind paw to the surgery; NSF, number of spontaneous flinches; PWMT, paw withdrawal mechanical threshold.

The PWMT and NSF were analyzed on days 0 (baseline), 4, 7, 10, 14, 21 after the operation to assess the mechanical hyperalgesia after sarcoma cell implantation. Significant differences in the PWMT and NSF of the ipsilateral hind paw were not observed between mice in the tumor and sham groups at baseline. On day 4 after surgery, a decreased PWMT and increased NSF were detected in both the tumor and sham groups of mice compared with the baseline values, which may result from the surgery. Nociceptive behaviors recovered beginning on day 7 and returned to the normal level on day 10 in the sham group of mice. Meanwhile, in the tumor group, the PWMT decreased and NSF increased persistently from day 10 to 21. Compared with the nociceptive behaviors in sham group, the behaviors in the tumor group showed significant differences on days 10, 14 and 21 after the operation. (figure 1A, B) In addition, the contralateral hind paw of all groups of mice showed no statistically significant differences in nociceptive behaviors. Therefore, BCP was successfully established on day 10 after the operation and continued to increase up to day 21.

A single intrathecal injection of JWH015 was administered on day 14 after the operation, and then the changes in the nociceptive behaviors of the ipsilateral hind paw over time in different groups were measured to explore the effect of CB2R activation on BCP. On day 14, all mice experienced pain, and a significant difference was not observed among the groups. After an injection of JWH015, an increasing trend in the PWMT and a decreasing trend in the NSF were observed. The antinociceptive effect of JWH015 appeared 2 hour after the injection, peaked at 12 hours and was sustained for 48 hours after the injection. However, nociceptive behaviors did not differ in the tumor+vehicle group and tumor+AM630+JWH015 group (figure 1C, D). Based on these data, treatment with JWH015 significantly reduced mechanical hyperalgesia in BCP mice.

### JWH015 inhibits the increase in BSCB permeability by increasing the levels of TJ proteins and protecting the structural integrity of capillaries

**Figure 2:**
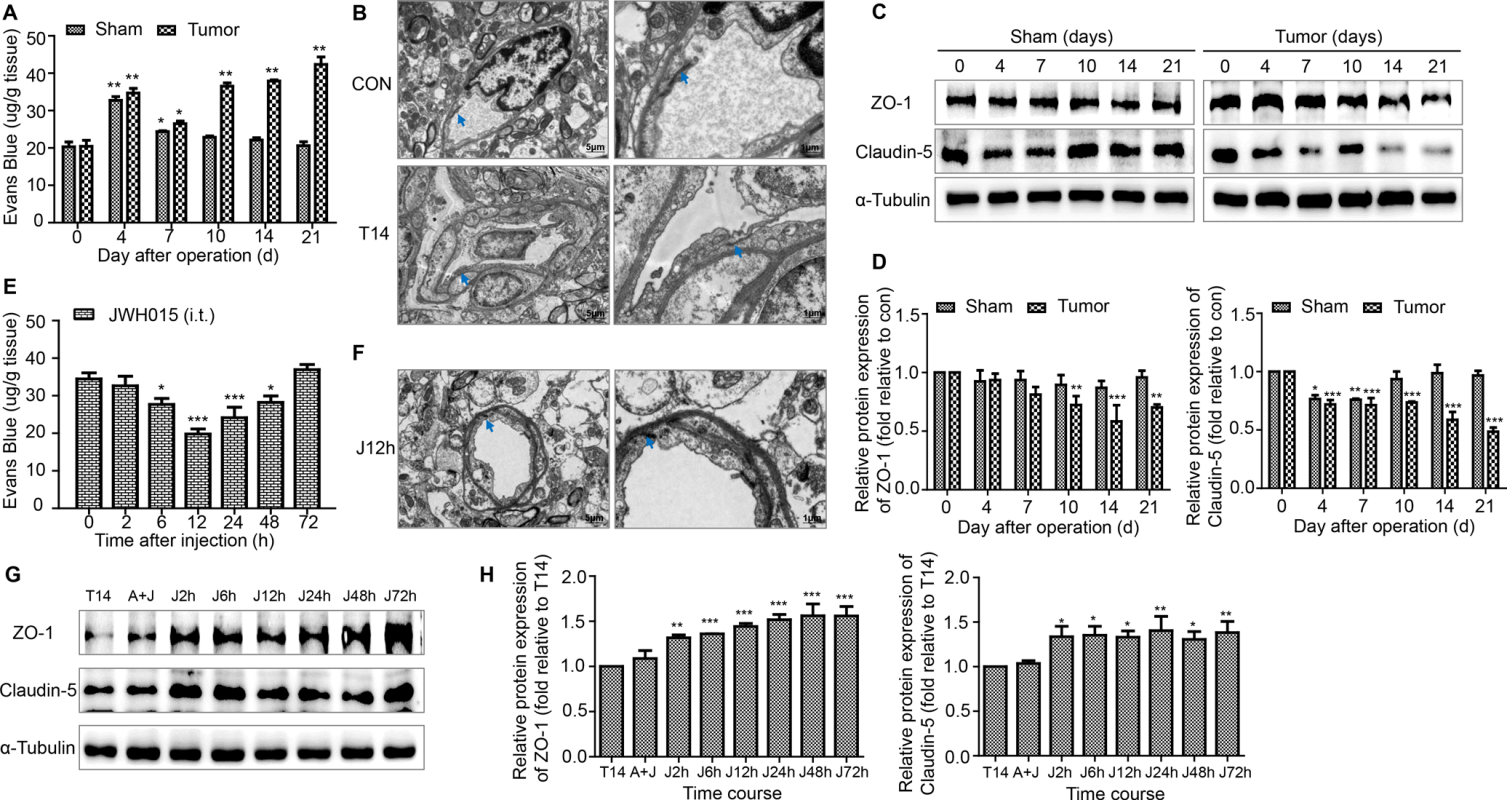
Disruption of the BSCB in BCP mice. (A) The EB leakage in the lumbar enlargement of spinal cord were tested on days 0, 4, 7, 10, 14 and 21 after operation in the sham and tumor groups. n=3 for each time point per group. (B) Representative images of the ultrastructure of the BSCB in the lumbar enlargement obtained using transmission electron microscopy on day 0 and day 14 in tumor group. Scale bar, 5 µm and 1 µm. n=3 per group. (C, D) Representative blots and quantification of TJ proteins ZO-1 and claudin-5 in the spinal cord on postoperative days in the sham and tumor groups. n=3 for each time point per group. *P<0.05 vs day 0, **p<0.01, ***p<0.001. Treatment of JWH015 improved the disruption of BSCB in BCP mice. (E) The EB leakage in the lumbar enlargement of spinal cord were tested before the injection (0 hour) and at 2, 6, 12, 24, 48 and 72 hours after the injection of JWH015 in the tumor group. n=3 for each time point per group. (F) Representative images of the ultrastructure of the BSCB in the lumbar spinal cord, which was taken from 12 hours after the injection of JWH015, obtained using transmission electron microscopy. Scale bar, 5 µm and 1 µm. n=3. (G, H) Representative blots and quantification of ZO-1 and claudin-5 at 2, 6, 12, 24, 48, and 72 hours after the injection of JWH015 compared with vehicle injection, respectively. n=3 for each time point per group. *P<0.05 vs vehicle injection, **p<0.01, ***p<0.001. All data are presented as the means±SD. Arrows showed TJ structures between the endothelial cells. BCP, bone cancer pain; BSCB, blood–spinal cord barrier; TJ, tight junctions.

A loss of BSCB integrity results in the leakiness of blood components, such as inflammatory mediators and activated immune cells, from the vessel into the spinal cord parenchyma. The Evans Blue extravasation assay was used to explore whether BSCB dysfunction participates in the development of BCP. An increase in the amount of Evans Blue dye that extravasated to the spinal cord parenchyma was observed on days 4 and 7 in both the tumor and sham groups of mice. From day 10 to day 21, a consistently remarkable increase in the amount of Evans blue dye was observed in the tumor group of mice, while the BSCB permeability returned to the normal level in the sham group of mice. Compared with the BSCB permeability in sham group, mice in tumor group showed significant differences on days 10, 14 and 21 after the operation (figure 2A). Furthermore, the amount of Evans Blue dye that extravasated was significantly reduced from 6 to 48 hours after treatment with JWH015 compared with the preinjection level (figure 2E).

Endothelial cells are the important components of the BSCB unit. We used electron microscopy to clearly observe the change in the BSCB structure. TJ between the endothelial cells were deformed and a gap was observed on day 14 in the tumor group compared with mice in the control group (figure 2B). Treatment with JWH015 significantly protected the structures of the BSCB. We chose 12 hours after the injection of JWH015 as the representative time point. The deformation of the TJ was improved (figure 2F).

TJ proteins located between endothelial cells ensure the tight structure of the BSCB. Next, we examined the alterations in the levels of the TJ proteins ZO-1 and Claudin-5 using western blotting. The level of ZO-1 decreased prominently from day 10 to 21 in the tumor group of mice, with no significant change in the sham group of mice. The level of claudin-5 decreased significantly on days 4 and 7 in both the tumor and sham groups of mice, consistent with the increase in BSCB permeability observed on days 4 and 7. From day 10 to 21, the level of claudin-5 continued to decrease further in the tumor group of mice, while it was restored to the normal level in the sham group of mice (figure 2C, D). JWH015 caused a continuous increase in the levels of ZO-1 and Claudin-5 from 2 to 72 hours after the injection compared with the preinjection values, and these changes were prevented by a pretreatment with AM630 (figure 2G, H).

Thus, JWH015 inhibited the increase in BSCB permeability by increasing the levels of TJ proteins and protecting the structural integrity of capillaries.

### JWH015 inhibits the activation of microglia and astrocytes in the spinal cord of BCP mice

**Figure 3:**
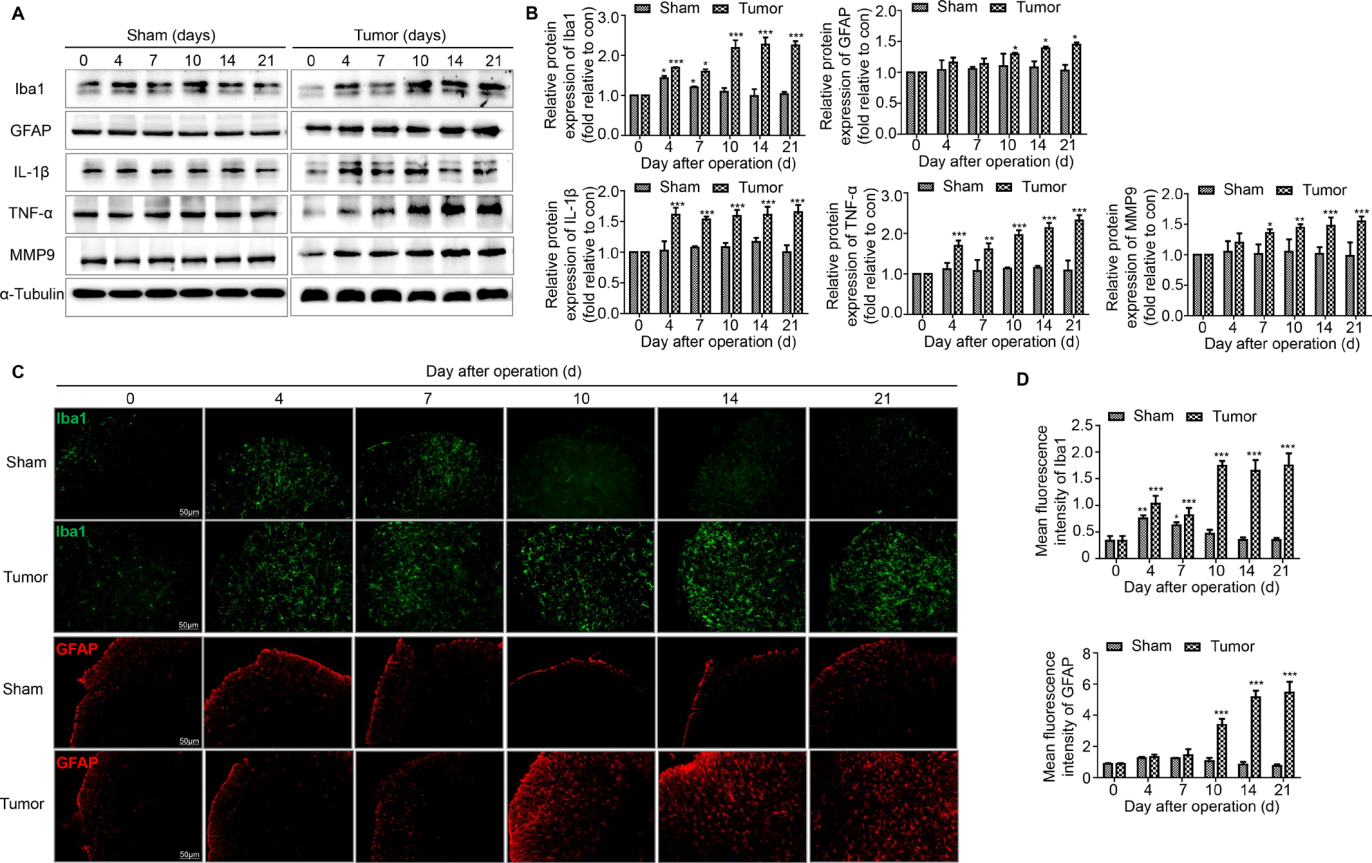
The activation of microglia and astrocytes and the expression of proinflammatory cytokines related to the disruption of BSCB in the spinal cord over time. (A, B) Representative blots and quantification of Iba1, GFAP, IL-1β, TNF-α and MMP9 on postoperative days in the sham and tumor groups. n=3 for each time point per group. (C, D) Immunofluorescent staining and quantification of mean fluorescence intensity of Iba1 (green) and GFAP (red) in the ipsilateral spinal cord dorsal horn of the sham and tumor group of mice over time. Scale bar, 50 µm. n=3 for each time point per group. *P<0.05 vs day 0, **p<0.01, ***p<0.001. All data are presented as the means±SD. BSCB, blood–spinal cord barrier; GFAP, a marker of astrocyte; Iba1, a marker of microglia; IL-1β, interleukin-1β; TNF-α, tumor necrosis factor α.

Hyperalgesia is usually accompanied by the activation of microglia and astrocytes in the spinal cord. The levels of microglial and astrocyte markers were measured during the development of BCP over time using western blotting and immunofluorescence staining. Iba1 was the marker of microglia and GFAP was the marker of astrocytes. In the tumor group of mice, Iba1 levels increased on day 4, decreased slightly on day 7 and increased significantly from day 10 to 21. Meanwhile in the sham group of mice, Iba1 levels was restored after day 10. In the tumor group of mice, GFAP levels increased significantly from day 10 to 21, but no obvious change was detected in the sham group of mice (figure 3A, B). The results of immunofluorescence staining were consistent with the western blotting data (figure 3C, D).

**Figure 4:**
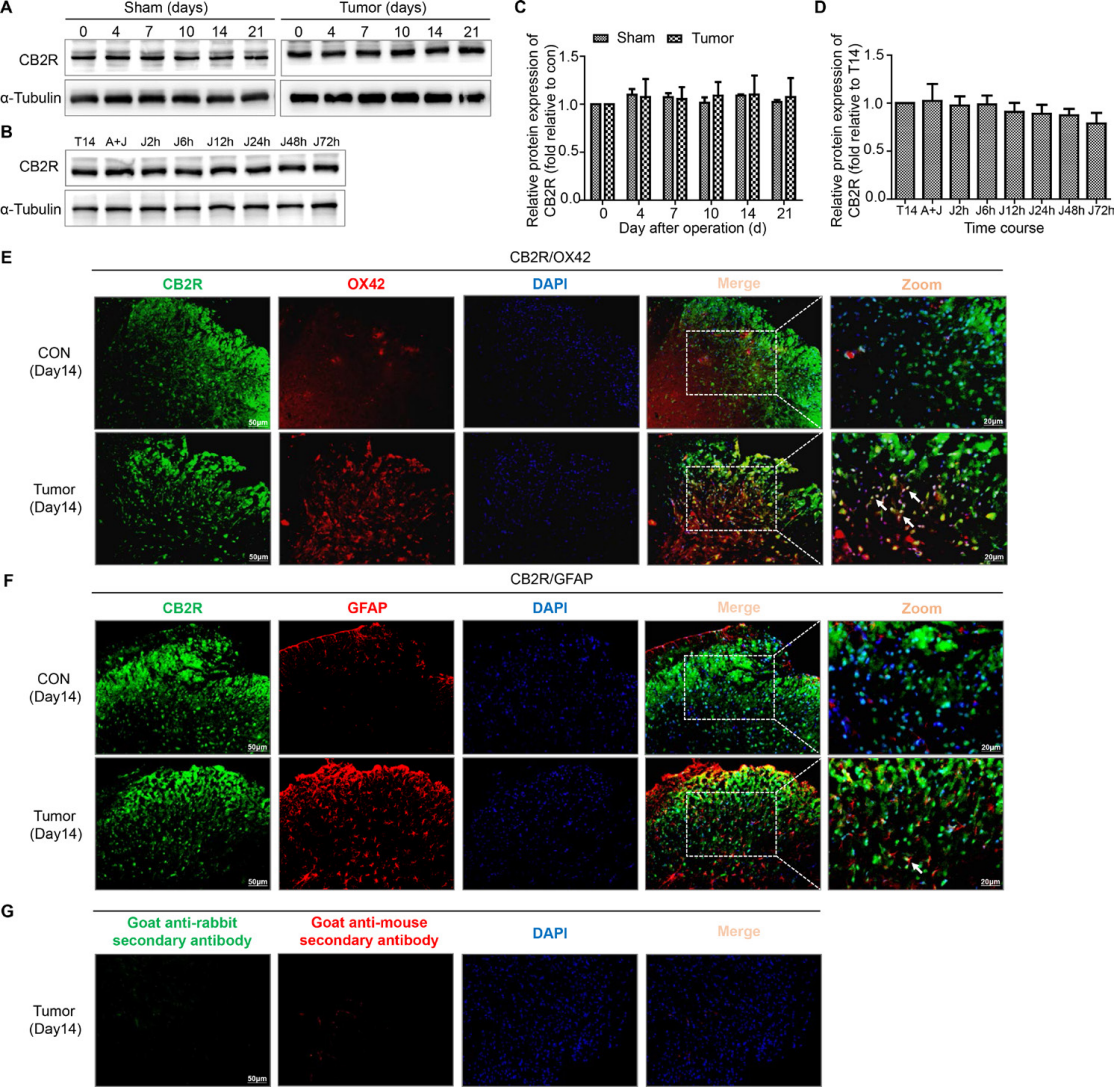
Colocalization of CB2R on microglia and astrocytes in the spinal dorsal horn of BCP mice. (A, C) Representative blots and quantification of CB2R on postoperative days in the sham and tumor groups. n=3 for each time point per group. (B, D) representative blots and quantification of CB2R at 2, 6, 12, 24, 48 and 72 hours after the injection of JWH015 compared with vehicle injection respectively. n=3 for each time point per group. (E) Double immunostaining of CB2R (green) and OX42 (red) in the spinal dorsal horn performing in Con and tumor (day 14) group mice. (F) Double immunostaining of CB2R (green) and GFAP (red) in the spinal dorsal horn performing in Con and tumor (day 14) group mice. Arrows showed the representative merge of CB2R and OX42 or GFAP. (G) Immunofluorescence negative controls. immunostaining of goat anti-rabbit secondary antibody (green) and goat anti-mouse secondary antibody (red) in the ipsilateral spinal dorsal horn of the tumor (day 14) group of mice. Scale bar, 50 µm and 20 µm. All data are presented as the means±SD. BCP, bone cancer pain; CB2R, cannabinoid receptor 2; GFAP, a marker of astrocyte; OX42, a marker of microglia.

We next assessed the expression and cellular localization of CB2R. The levels of CB2R were not obviously altered in both sham and tumor group of mice (figure 4A, C). The results of double immunofluorescence staining showed that CB2R was mostly colocalized with OX42 (a microglia marker) and a few with GFAP in the spinal dorsal horn (figure 4E, F).

**Figure 5:**
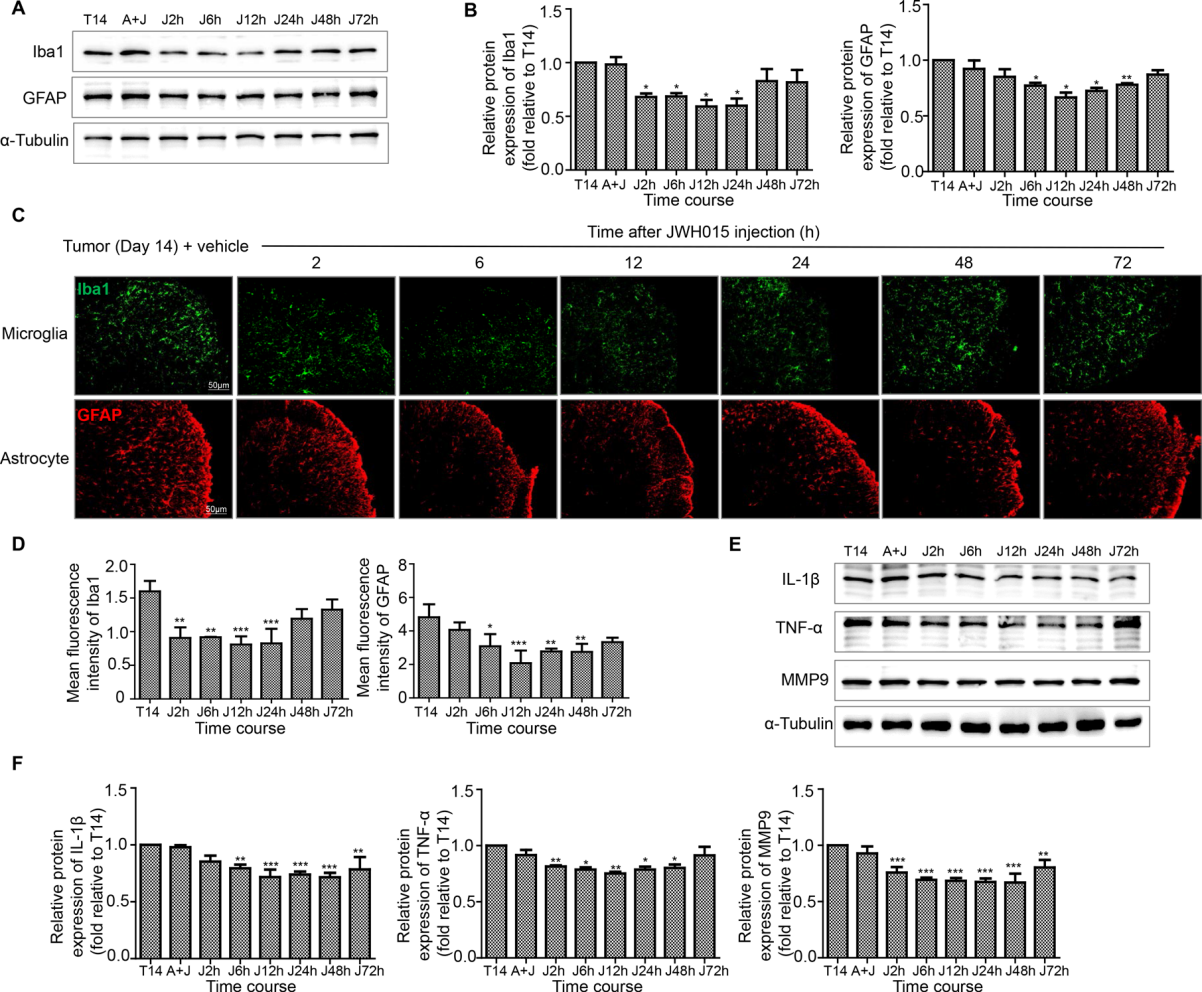
Treatment of JWH015 decreased the activation of microglia and astrocytes and the expression of proinflammatory cytokines related to the disruption of BSCB in the spinal cord over time. (A, B) Representative blots and quantification of Iba1 and GFAP at 2, 6, 12, 24, 48 and 72 hours after the injection of JWH015 compared with vehicle injection, respectively. n=3 for each time point per group. (C, D) Immunofluorescent staining and quantification of mean fluorescence intensity of Iba1 (green) and GFAP (red) in the ipsilateral spinal cord dorsal horn at 2, 6, 12, 24, 48 and 72 hours after the injection of JWH015 compared with vehicle injection, respectively. Scale bar, 50 µm. n=3 for each time point per group. (E, F) Representative blots and quantification of IL-1β, TNF-α and MMP9 at 2, 6, 12, 24, 48 and 72 hours after the injection of JWH015 compared with vehicle injection respectively. n=3 for each time point per group. *P<0.05 vs vehicle injection, **p<0.01, ***p<0.001. All data are presented as the means±SD. BSCB, blood–spinal cord barrier; GFAP, a marker of astrocyte; Iba1, a marker of microglia; IL-1β, interleukin-1β; TNF-α, tumor necrosis factor α.

After treatment with JWH015, lumbar enlargements collected from mice at different points were processed for immunostaining and western blotting to determine whether the protective effect of JWH015 on the BSCB was mediated by the inhibition of microglial and astrocyte activation. The level of CB2R was not obviously altered after the injection of JWH015 (figure 4B, D). As shown in the representative micrographs of immunostaining and the quantitation of the mean fluorescence intensity, microglial activation was significantly inhibited from 2 to 24 hours after the injection of JWH015, began to recover at 48 hours and returned to the preinjection level at 72 hours compared with mice in the tumor+vehicle group. The activation of astrocytes was significantly inhibited from 6 to 48 hours after the injection of JWH015 (figure 5C, D). Western blotting analyzes showed a similar decrease in the levels of Iba-1 and GFAP, which were consistent with the result of the immunofluorescence staining (figure 5A, B). The inhibitory effect was abolished after a pretreatment with AM630. Based on these data, JWH015 inhibited the activation of microglia and astrocytes in BCP mice.

### JWH015 suppresses the release of proinflammatory cytokines related to the disruption of the BSCB in the spinal cord

The activation of microglia and astrocytes would induce the release of large amounts of proinflammatory cytokines related to the disruption of BBB. We detected the levels of three key factors in the tumor group of mice: IL-1β, TNF-α and MMP9. Consistent with the disruption of the BSCB, remarkably increased levels of IL-1β, TNF-α and MMP9 were observed in the tumor group of mice. IL-1β and TNF-α levels increased significantly from day 4 to day 21. MMP9 levels increased significantly from day 7 to day 21. The levels of these proinflammatory factors were not obviously altered in the sham group of mice (figure 3A, B).

The time course of the effects of JWH015 on the production of inflammatory factors was investigated. The levels of IL-1β, TNF-α and MMP9 were decreased by the injection of JWH015 compared with the injection of vehicle. Meanwhile, no corresponding changes were observed in mice injected with AM630. In detail, compared with the preinjection level, IL-1β levels decreased from 6 to 72 hours after the injection of JWH015, TNF-α levels decreased from 2 to 48 hours after the injection, and MMP9 levels continuously decreased from 2 to 72 hours (figure 5E, F). Thus, JWH015 inhibited the release of proinflammatory mediators related to the disruption of the BSCB.

## Discussion

In our study, proinflammatory factors produced from activated microglia and astrocytes acted on the endothelial cells, contributing to the sustained disruption of the BSCB and persistent BCP. A single intrathecal injection of JWH015 actually protected the BSCB from damage and alleviated pain hypersensitivity by inhibiting the activation of microglia and astrocytes, and decreasing the expression of IL-1β, TNF-α and MMP9.

BCP remains a challenging problem in the clinic due to its complex and poorly elucidated etiology and mechanisms.^21^ BCP is a mixture of ongoing inflammatory and neuropathic processes that lead to peripheral and central sensitization.^1^ The current therapeutic options for BCP are not very effective and have many unresolvable side effects.^22^

Endocannabinoids play important roles in regulating analgesia. Based on our results, effective analgesia was achieved, as described in models of neuropathic pain,^23^ remifentanil-induced hyperalgesia^5^ and cancer-induced pain.^18^ In the present study, treatment with JWH015 alleviated pain hypersensitivity in a time-dependent manner. The antinociceptive effect of JWH015 appeared at 2 hours, peaked at 12 hours and was sustained for 48 hours after the injection. Many possible mechanisms may underlie the analgesic effect, including inhibiting the hyperactivation of spinal glial cells and production of proinflammatory cytokines, inhibiting the phosphorylation of NR2B in the spinal cord. However, little attention has been paid to BSCB damage.

Under physiological conditions, the BSCB represents a tight barrier between the blood and CNS. Disruption of the BSCB occurs under various pathological conditions, such as ALS,^24^ spinal cord injury^25^ and neuropathic pain,^10^ leading to increased permeability and subsequent damage. As shown in our previous study, T cells infiltrated into the spinal cord during the maintenance of BCP.^26^ Therefore, we proposed the hypothesis that BSCB disruption contributed to the maintenance of BCP. In this study, we observed a persistent increase in the permeability of the BSCB during the development of BCP from day 10 to 21, however, it returned to the normal level in the sham group of mice. Meanwhile, transmission electron microscopy identified the disruption of BSCB ultrastructure in the tumor group of mice. Furthermore, the levels of ZO-1 and claudin-5 decreased significantly during the maintenance of BCP, with no significant changes in sham mice. Therefore, we postulated that the disruption of the BSCB was responsible for the progressive BCP.

A relationship between CB2R and the BBB has been proposed in various diseases. The activation of CB2R potentially attenuates BBB damage in encephalitis,^27^ a rat ICH model^28^ and spinal cord ischemia-reperfusion injury.^29^ We wanted to determine whether JWH015 protected the BSCB in a BCP model. We detected the permeability of the BSCB after the injection of JWH015. Treatment with JWH015 significantly improved the permeability compared with the preinjection observation, consistent with the nociceptive behaviors. Treatment with JWH015 significantly protected the ultrastructure of the BSCB. Furthermore, ZO-1 and claudin-5 levels were increased significantly after the injection of JWH015. ZO-1 and claudin-5 are representative, but not unique, markers of the structural integrity of the BSCB. Thus, a certain degree of difference between protein expression and changes in the BSCB permeability may occur after administration. Overall, the analgesic effect of JWH015 on BCP may be mediated by the protection of the BSCB.

We focused on neuroinflammation in the spinal cord to explore the mechanism by which JWH015 improved the function of the BSCB. Proinflammatory cytokines such as IL-1β and TNF-α and the enzyme MMP9 play important roles in BSCB disruption. IL-1β alters the endothelial permeability in the brain of subjects with neuropathic pain.^30^ TNF-α-induced endothelial permeability in vitro and is associated with an increase in the BSCB permeability in peripheral neuropathic injuries.^11^ MMP9 mediated an increase of BSCB permeability by disrupting TJ proteins during ischemic spinal cord injury.^31^ IL-1β, TNF-α and MMP9 can be produced and released by overactivated microglia and astrocytes.^15 16^ We observed the hyperactivation of glial cells in the BCP rat model in our previous study, but did not examine MMP9.^26 32^ In the present study, the activation of microglia and astrocytes and the levels of IL-1β, TNF-α and MMP9 in the spinal cord increased significantly during the maintenance of BCP, but recovered to normal levels in sham mice. Thus, the proinflammatory factors that are produced and released by activated glia might underlie the disruption of BSCB during the maintenance of BCP. In addition, the activation of microglia and the increases in the levels of IL-1β and TNF-α on days 4 and 7 may result from the surgery and contribute to the short-term BSCB damage. Our results were consistent with the findings reported by Smith *et al* that BSCB permeability increases transiently only after peripheral neuropathic injury.^11^

The CB2R is expressed at high levels in the mouse brain, including mainly microglia and astrocytes, some subpopulations of neurons and endothelial cells.^8^ In the present study, CB2R mainly located on microglia and a few on astrocytes in the spinal dorsal horn of the BCP model. The levels of activated microglia and astrocytes and the levels of IL-1β, TNF-α and MMP9 were detected at various time points after the injection to elucidate the mechanism by which JWH015 protected the BSCB. The activation of microglial and astrocyte was significantly inhibited after the injection of JWH015. The levels of IL-1β, TNF-α and MMP9 also decreased significantly after the injection. However, all of the effects of JWH015 were blocked by the CB2R antagonist AM630. These findings suggest that JWH015 reverses BSCB damage during BCP by inhibiting inflammation produced by activated glia.

Although the CB2R agonist has been shown to function as an analgesic in several types of pain models, we are not aware of any study that has explored the effect of the CB2R agonist on the integrity of BSCB in BCP. Furthermore, we have explored the efficacy and mechanisms of JWH015, a CB2R-selective agonist, in several pain models. As shown in our previous study, an intraperitoneal injection of JWH015 reduces hyperalgesia in a rat BCP model, similar to intrathecal injection at certain doses.^33^ Since JWH015 is a lipophilic small molecule, it should have access to the spinal cord. Thus, JWH015 has clinical therapeutic potential in treating BCP. Therefore, studies aiming to explore new analgesic mechanisms of JWH015 are needed.

All the data presented in the present study suggest the possible pathway in which JWH015 protected the damaged BSCB and alleviated BCP by decreasing the level of proinflammatory factors produced and released from activated microglia and astrocytes, but other mechanisms of action should not be excluded. For example, JWH015 may directly bind to CB2R located on the BSCB endothelial cells to promote TJ formation and reduce the infiltration of circulating inflammatory cells and cytokines into the spinal cord.

Estrogen and estrogen receptor play important roles in the development of BCP.^34^ Based on accumulating preclinical evidence, estradiol may alter the CB1R density, affinity and function, as well as the endocannabinoid content.^35^ We are not aware of any studies exploring the effect of estradiol on CB2R or any studies comparing spinal cannabinoid antinociception between males and females of any species. We used male mice in our study to exclude the possible effect of estrogen.

## Conclusions

In conclusion, CB2R activation mediated by a single intrathecal injection of the agonist JWH015 relieves hyperalgesia associated with BCP. The antihypersensitivity effect of spinal CB2R activation correlates well with the improvement in the BSCB integrity. Based on our data, spinal CB2R activation likely relieves BCP by minimizing the disruption of the BSCB.
